# Obsolete tobacco control themes can be hazardous to public health: the need for updating views on absolute product risks and harm reduction

**DOI:** 10.1186/s12889-016-3079-9

**Published:** 2016-05-24

**Authors:** Lynn T. Kozlowski, David B. Abrams

**Affiliations:** Department of Community Health and Health Behavior, University at Buffalo, State University of New York, Buffalo, NY 14214-8028 USA; The Schroeder Institute for Tobacco Research and Policy Studies, Truth Initiative, Washington, DC USA; Department of Health, Behavior and Society, Johns Hopkins Bloomberg School of Public Health, Baltimore, MD USA; Department of Oncology, Lombardi Comprehensive Cancer Center, Georgetown University Medical Center, Washington DC, USA

**Keywords:** Tobacco policy, Smoking, Smokeless tobacco, Electronic cigarettes, Harm reduction, Vaping, Snus, Nicotine, Risk communication, Warning labels

## Abstract

**Background:**

Leading themes have guided tobacco control efforts, and these themes have changed over the decades. When questions arose about health risks of tobacco, they focused on two key themes: 1) how bad is the problem (i.e., absolute risk) and 2) what can be done to reduce the risk without cessation (i.e., prospects for harm reduction). Using the United States since 1964 as an example, we outline the leading themes that have arisen in response to these two questions. Initially, there was the recognition that “cigarettes are hazardous to health” and an acceptance of safer alternative tobacco products (cigars, pipes, light/lower-tar cigarettes). In the 1980s there was the creation of the seminal theme that “Cigarettes are lethal when used as intended and kill more people than heroin, cocaine, alcohol, AIDS, fires, homicide, suicide, and automobile crashes combined.” By around 2000, support for a less-dangerous light/lower tar cigarette was gone, and harm reduction claims were avoided for products like cigars and even for smokeless tobacco which were summarized as “unsafe” or “not a safe alternative to cigarettes.”

**Discussion:**

The Surgeon General in 2014 concluded that by far the greatest danger to public health was from cigarettes and other combusted products. At the same time the evidence base for smokeless tobacco and alternative nicotine delivery systems (ANDS) had grown. Product innovation and tobacco/nicotine bio-behavioral, epidemiological and public health sciences demonstrate that low nitrosamine smokeless tobacco (e.g., Swedish snus), and ANDS have substantially lower harms than cigarettes. Going forward, it is important to sharpen themes and key messages of tobacco control, while continuing to emphasize the extreme lethality of the inhaled smoke from cigarettes or from use of any combusting tobacco product.

**Summary:**

Implications of updating the leading themes for regulation, policymaking and advocacy in tobacco control are proposed as an important next step. A new reframing can align action plans to more powerfully and rapidly achieve population-level benefit and minimize harm to eliminate in our lifetime the use of the most deadly combustible tobacco products and thus prevent the premature deaths of 1 billion people projected to occur worldwide by 2100.

## Background

### Leading themes

For much of the 20^th^ Century, cigarette smoking was for many a socially acceptable, even fashionable activity with considerable social pressure on some individuals to become smokers [1, 2]. By the end of that century, cigarette smoking had been recognized as a major cause of premature death and disability, [3] and health authorities from around the world had mobilized to stop the public health tragedy of tobacco use [4]. When questions arose about the ill-effects of a very popular product like tobacco, they usually focused on two key themes: 1) how bad is the problem (i.e., the absolute risk) and 2) what can be done to reduce the risk without giving up such products (i.e., the prospects for harm reduction [5]). Using the United States (U.S.) as an example, we outline the leading themes that have arisen in response to these two questions. Both the issues of absolute risk and harm reduction have continuously been present, but perspectives have changed significantly. This is not a review of epidemiological results, but a consideration of these leading themes, which provide a view of changing emphases in tobacco control.

Although the history of societal responses to tobacco use is centuries old, [1] the 1964 publication of “Smoking and Health: Report of the Advisory Committee to the Surgeon General on Smoking and Health” [6] marks a starting point for concerted tobacco control efforts in the U.S.. At every point since 1964, tobacco control has had a dominant view of both absolute risk and tobacco harm reduction. Table 1 provides an overview and summarizes some of the variations in views on these two themes.

**Table 1:** Timetable of leading tobacco control themes in the United States on absolute risk and harm reduction options

Approximate dates in United States	Leading Absolute Risk Theme	Harm Reduction
1964 -	Cigarettes are hazardous to men and likely to be for women.	Cigars and pipes are safer than cigarettes; lower-tar cigarettes may be safer; the science that put a man on the moon will develop a safer cigarette soon.
1980-	Cigarettes are lethal when used as intended and kill more people than heroin, cocaine, alcohol, AIDS, fires, homicide, suicide, and automobile accidents combined.	Cigars and pipes are safer than cigarettes; Light/lower-tar cigarettes may be safer; Snus is less harmful (in Sweden/Scandinavia).
1987-	Cigarettes are lethal (as above) and cause lung cancer, heart disease, emphysema; smokeless tobacco and cigars are not safer alternatives to cigarettes.	Avoidance of indication of harm reduction from cigars and smokeless tobacco; tar and nicotine testing stopped, but “low-tar” and “Light” claims still marketed and misleading.
2001-	All cigarettes are equally lethal; all tobacco products are unsafe.	No recognition/encouragement of less-harmful tobacco use.
2014-	“The burden of death and disease from tobacco use in the United States is overwhelmingly caused by cigarettes and other combusted tobacco products....”	An acknowledgement of the special deadliness of the smoke from combustion, primarily from smoking cigarettes and the potential for harm reduction.
	All cigarettes are equally lethal; all tobacco products are unsafe.	
2015- [Herein Proposed]	Cigarettes and other smoked products are the most deadly; non-combusted tobacco and alternate nicotine delivery products, including medical replacement therapies are unsafe, but relatively low in risk. Smoke for the nicotine but die from the tar.	An acknowledgement of the special deadliness of smoking and development of ways to increase harm reduction in continuing users of lethal tobacco products by displacing smoking with much less harmful tobacco or nicotine.

### “Cigarettes are hazardous to your health,” but you have options

The 1964 Surgeon-General’s Report had considerable impact on media reports, organized action, and on the American public [7]. It can be underappreciated that at the time, the report raised concerns about a major, then respected industry. There were earnest hopes that the risks of cigarettes could be reduced, and lower-risk options were noted. For example, the conclusions for lung cancer read:

Cigarette smoking is causally related to lung cancer in men; the magnitude of the effect of cigarette smoking far outweighs all other factors. The data for women, though less extensive, point in the same direction....

The risk of developing cancer of the lung for the combined group of pipe smokers, cigar smokers, and pipe and cigar smokers, is greater than for non-smokers, but much less than for cigarette smokers. The data are insufficient to warrant a conclusion for each group individually (Chapter 9, p. 196).” [p.37]

In addition to efforts to prevent and treat cigarette smoking, there was advice from the Surgeon General, the President of the American Medical Association, and the Consumers Union that switching from cigarettes to cigars or pipes was a useful option for those who would not quit tobacco completely, and the sales of cigars boomed [8]. Lung cancer expert Ernst Wynder at the Sloan-Kettering Cancer Institute reported in *Life Magazine* that the technology that had put a man on the moon could be used to make safer cigarettes [9]. Days before the 1964 Surgeon-General’s Report was released the first very-low-tar yield (so called, “light”) ventilated-filter cigarette was released—with tar and nicotine yields printed on the pack [9]. The sales of filtered, light/lower-tar cigarettes boomed, and the government started testing cigarettes for tar and nicotine yields in 1967; a lower-tar race was underway [9]. In Senate testimony in 2007, the Federal Trade Commissioner reported that in 1967, “most public health officials believed that reducing the amount of ‘tar’ in a cigarette could reduce a smoker’s risk of lung cancer; therefore, it was thought that giving consumers uniform and standardized information about the tar and nicotine yields of cigarettes would help smokers make informed decisions about the cigarettes they smoked [10].” The National Cancer Institute, in collaboration with the cigarette industry, actually undertook research to help develop less-hazardous cigarettes [11].

In retrospect, the aggressive, optimistic acceptance of cigarette harm reduction was a tragedy. The disaster of the light/low-tar cigarette was compounded by the product’s popularity and the fact that risks were not meaningfully reduced though the perception was that they had been reduced [12]. It would take decades of research to prove that lower-tar cigarettes were not worthwhile reduced-harm products [13] and to understand that inhaled cigar and pipe smoke (inhalation was more likely in former cigarette smokers) were significantly dangerous to health [14]. The recognition was yet to come that it was the toxic inhaled smoke from the combusting of tobacco (the mode of delivery) that carried the greatest harm (cigarettes, cigars, pipe and roll your own and hookah).

The history of cigarette warning labels in the U.S. has been described in detail [15]. In 1966, cigarette packages only (not advertising) were required to have the warning: “Caution: Cigarette Smoking May Be Hazardous to Your Health.” This is a warning that lives in infamy as a tragically cautionary stance. In 1970, cigarette advertising was banned on television and radio, and the package warning was strengthened to: “Warning: The Surgeon General Has Determined That Cigarette Smoking Is Dangerous to Your Health.” By 1985, warnings included indication that cigarettes caused lung cancer, heart disease, and emphysema. In 1973, the ban on broadcast advertising was extended to little cigars [15].

### Cigarette control in the 1980s: the extraordinary dangers of smoking

In the 1980s, “cigarette control” forces were learning new ways to battle the powerful public relations/marketing symbols that the industry employed in defense of their products. Broader advertising bans on cigarettes were being proposed in the U.S. [16] and elsewhere [17–19]. The industry argued that a ban for cigarettes would be a “slippery slope” or the “thin edge of the wedge,” and that other, popular unsafe products (alcohol, cars, salt, butter, and fat) would be next in line for advertising bans and other meddlesome regulatory constraints. A group of international “cigarette control” experts from 35 countries were convened in 1985 by the American Cancer Society, and as a result, Michael Pertschuk led the development of an influential advocacy guide [20]. This “smoking control media handbook” reproduced the industry’s arguments and gave birth to symbolically powerful responses that reframed the issues.

- The degree of regulation appropriate for alcohol and alcohol advertising is a debatable point which is resolved by each society as it sees fit, balancing both the serious social and health hazards of alcohol, the ability of most users to maintain moderate, safe levels of consumption, and evidence that the moderate use of alcohol is not a health threat for many people.
- Automobiles involve serious risk, but they are indispensable to modern society, and the risks are substantially reduced when cars are engineered safely and appropriate traffic laws are enacted, enforced and obeyed.
- Fat, sugar, and salt are essential to life and become hazardous only when consumed in excess.
- **Cigarettes are the only legal product that, when used as intended, are lethal.** [Emphasis added.]
- **Smoking is not only a hazard to the smoker, but also to the nonsmoker who is involuntarily exposed to the smoke. Consuming fat, sugar, and salt is not a hazard to bystanders.** [Emphasis added.]
- **Smoking kills more people than heroin, cocaine, alcohol, AIDS, fires, homicide, suicide and automobile accidents COMBINED.** [Emphasis added.]

Cigarette control experts used some variant of this three-pronged argument to reframe their strategy: 1) “cigarettes are the only legal products that are deadly when used as intended by the manufacturer,” 2) involuntary smoking is a unique concern, and 3) the deadliness of cigarettes is extreme (i.e., defective) and much greater than for other products [21].

The force of these arguments was critical to the “de-normalization” of cigarettes and to the institution of cigarette control measures (increased taxation, clean indoor air laws) that have contributed to the decrease in cigarette prevalence. The phrasing “harmful when used as intended by the manufacturer” has a long history in product liability law and government regulations. (To appreciate why this principle got nowhere in relation to legal tobacco liability issues, see these accounts [22, 23]). The *Smoke Signals* handbook helped popularize the framing of these arguments for cigarettes being different from other popular, harmful products (like alcohol).

This theme helped distance cigarette control advocates from those who wanted to restrict marketing for other unsafe products which, although not without some risk to individual users, were much less dangerous than cigarettes on a population-wide basis. In the U.S., society and regulators have accepted quite open marketing for a number of sometimes popular, unsafe products (e.g., alcohol, acetaminophen, prescription medications) [24]. At the time, the cigarette control field understood that claiming a product was unsafe was the beginning of an argument, not the end of the argument, on how a product should be marketed.

Consistent with the unintended consequences of the unfortunate theme reported in *Life Magazine* that the technology that had put a man on the moon could be used to make safer cigarettes [9] and the subsequent misguided entrusting of the industry to work with government to develop safer cigarettes, the greatest fraudulent claims of the light/low tar cigarette era was born. The 1980s still represented a boom time for light/lower-tar cigarettes. These ventilated-filter products made up the large majority of cigarette sold, and they were mistakenly perceived as less dangerous than higher tar cigarettes by many consumers aided by Government testing and labeling where the unscrupulous industry found ways to cheat the Federal Trade Commission (FTC) testing method until testing was stopped [25, 26].

### ‘Cigarette control’ becomes ‘tobacco control’ in 1980s: Smoking is bad and there are no product options to consider

In 1987, rotating warnings were finally added to packages and advertising of smokeless: “WARNING: This product may cause mouth cancer,” “WARNING: This product may cause gum disease and tooth loss,” and “WARNING: This product is not a safe alternative to cigarettes.” Despite the substantive contrast between the warnings (and the evidence base) for cigarettes and smokeless tobacco, public health messages in the 1980 and 1990s often stressed that smokeless tobacco was not safer than cigarettes. This theme essentially blurred the distinction between combustion and non-combustion in ironic contrast to the encouragement to switch to pipe and cigars made by experts in 1964 [8]. For example, the Surgeon-General’s Report for Kids, when asking “If smokeless tobacco was “safer’ than cigarettes,” answered “NO WAY!”, even though their elaboration of that point (and the official warning) made clear that the health problems were really quite different from those known for cigarettes. See discussion of this in [27, 28]. Perhaps as a strong backlash to the fraudulent behavior of an untrustworthy industry (i.e. the light/low tar debacle) the pendulum understandably swung to the other extreme. The data that not all tobacco products were equally harmful (i.e., smoked verus smokeless) was overshadowed by the trusim that there was some harm caused by all tobacco use. Thus the harm reduction baby was thown out with the bathwater and a major swing to an all or nothing stance was adopted. A justified rage and misrust of the industry led to a emergent prohibitionist theme. One exception was arising in the Scandanavian countries especially Sweden in the late 1970’s with the introduction in Sweden of a moist form of Swedish snus and a voluntary standard (Gothiatek) to produce and market a low nitrosamine form of smokeless tobacco. However, this recognition of harm reduction was ignored and met with suspicion and hostility by the rest of the world (see more detailed discussion below).

It is important to distinguish between products that are “not safe” and those that are “not safer” than cigarettes. Special efforts were made to discourage smokeless tobacco as a safer alternative to cigarettes, and one should assume that, if the claim “Not Safer than cigarettes” was scientifically defensible, it would have been made in the official warnings. This distance between not safe and deadly can be large, and it is a very limited contribution to health communication to say a product is “not safe” with no indication of the level of absolute or relative risk of harm [28].

“Cigarette control” was subsumed by “tobacco control,” and such an incorporation contributed to a blurring or conflation of issues across classes of products that are in fact quite different from cigarettes in the damages caused. Given the dangers of consumer products (e.g., alcohol, a number of prescription and non-prescription pharmaceuticals, automobiles, processed meats), one should be reluctant to use the argument on the extreme deadliness of cigarettes to oppose the use of other products that, while not safe, will likely be or have been shown to be substantially less dangerous than cigarettes. Understanding the complex systems forces at work that have blurred the cigarette/combustible tobacco focus as the prime cause of preventable death is a challenge. The basic cigarette/combustible class of product has been “improved” (in appeal, addiction liability and toxins) but is largely unaltered and has dominated sales for over 120 years, a period aptly termed the “Cigarette Century” and more recently the “Golden Holocaust.” [2, 29]. By 1987 the FTC stopped doing machine-smoked tar and nicotine tests [10]. By 2001, the hope for a reduced-risk combusted cigarette seemed officially gone [13]. We turn now to a discussion of the dramatically changing landscape and the need to further sharpen the important themes going forward in tobacco control.

## Discussion

### Understanding and managing differential risks of alternative nicotine delivery products (ANDS), non-combusted tobacco products, and combusted tobacco products

Since 1964, major themes missed a core principle: The substantially greatest harm is from the toxic smoke of combusted, inhaled tobacco. In a 2014 summary of 50 years of research on tobacco and health, the U.S. Surgeon General finally concluded, “The burden of death and disease from tobacco use in the U.S. is overwhelmingly caused by cigarettes and other combusted tobacco products....” [30]. This opened the door to an evidence-based re-depolyment of harm reduction in tobacco control, but there have been challenges to getting the field to step through that door. Some of the challenge arises, we think, because of a mistaken but understandable lumping of all tobacco/nicotine products into the same bin of being highly lethal when used as intended and more dangerous than an array of other unsafe products and activities. Tobacco control needs to be guided by a modern understanding of differential risks from different modes of delivery of tobacco/nicotine containing products in the practice of tobacco control, not crude, unjustified claims of product risks based on the fraudelent industry behavior of the light/low tar disaster.

The tobacco and nicotine delivery marketplace has changed dramatically with three landmark developments: (a) introduction and acceptance of alternative forms of medicinal nicotine replacement therapies (NRT’s) for smoking cessation deemed safe for over-the-counter sales and for long-term use if need be; (b) the recent introduction and promise of future improved innovation of the disruptive technologies of a range of alternative nicotine delivery systems (ANDS) such as disposable, tank and Mod vapor products (e-cigarettes); and (c) rigorous and convincing longitudinal epidemiological data from Sweden/Scandinavia of the successful use of low nitrosamine Swedish snus for harm reduction.

Tobacco control is at a critical crossroads. Issues of absolute risks and harm reduction options have become divisive in the science, practice and policy [31, 32] arenas as the marketplace changes and as old status quo arguments are questioned. The rhetoric and argumentation arising from smoking (i.e. combustible products: primarily cigarettes, cigars, pipes, roll your own and hookah) and health does not translate well to the substantially less harmful classes of products: smokeless tobaccos and various emerging electronic cigarette innovations for nicotine aerosol inhalation (vape), or other ANDS, that, like NRT’s, de-couple nicotine delivery from the complex lethal toxins of tobacco combustion.

The 2014 Surgeon-General’s report [30] encourages a new framework in tobacco/nicotine control. The dominant argument against *a product that was lethal when used as intended and more deadly than a list of dangerous products* applies to cigarettes in particular and the toxic inhaled smoke from combustible tobacco products. It is frankly unlikely that this argument fits at all for vaping (aerosol delivery of nicotine in a humectant, without the carbon monoxide, over 4.000 chemicals and the extreme levels of harm from the over 60 known human carcinogens in deadly smoke) or smokeless tobacco, especially the low nitrosamine forms produced in Swedish type snus. The toxicological and epidemiological evidence pertaining to harms from these products is very different than for combustibles [33–35]. There are many reasons for discouraging the use of several popular consumer products, especially when it comes to preventing youth initiation of any and all forms of nicotine delivery systems (NRT or ANDS) or tobacco products, regardless of their differential harm profiles. However, it is now crystal clear that it is the inhaled deadly smoke from cigarettes/combustibles that stands alone by orders of magnitude as a pinnacle of deadliness that greatly exceeds the disease and disability costs of a large number of consumer products added together as well as NRT, ANDS and all forms of non-combusted tobacco [36, 37].

A view that treats all tobacco/nicotine use as equally bad is no longer consistent with the evidence base and represents a runaway rhetoric. Given the relative risks of different classes of tobacco/ANDS products, one should not let a broad commitment to “tobacco control” distract from the most important goal of cigarette/combustible smoking elimination. Those who have come to treat all tobacco/nicotine products as equally repugnant would have an expected resistance to any loosening of the dominant themes and frameworks appropriate to the prior 50 years of the tobacco product and control landscape. Given the disruptive technological innovations of the last 5 years, one can expect a new period of uncertainty and strong emotion as old foundational assumptions, fears and justifiable tobacco industry mistrust is stirred up.

Nonetheless, the new reality of ANDS, smokeless/snus and NRT’s must be fully recognized and thus, there is an urgent critical need for old views to be re-examined, some retained, others set aside (some prior tried and true past views may now in fact be counterproductive or destructive) and new frameworks developed to fit the new emerging scientific evidence and the evolving and rapidly transforming landscape of alternative nicotine modes of delivery in the marketplace [31, 32, 38–40]. We see the current turmoil as an understandable loosening of prior views, and the chaos is inevitable as it portends a new synthesis or systems integration--described so aptly by Kuhn in the history of scientific revolutions from Ptolemy to Copernicus to Einstein [41]. The 120+ year dominance of the “cigarette century” ushered in with the disruptive technology of the cigarette rolling machine in 1882 is being seriously challenged, perhaps for the first time in 140 years, by the emergence of newer and much less harmful modes of nicotine delivery, and is explored in detail elsewhere: [42] First by the introduction of medicinal nicotine therapy, [32] second by evidence of low harm smokeless tobacco in Sweden/Scandinavia, [43] and third by emergence of disruptive technological innovations in aerosolized nicotine delivery (e.g. vaping of e-cigarettes) without any tobacco per se [31, 32, 42].

### Is snus or ANDS more lethal than any of these separately: heroin or cocaine or alcohol or AIDS or fires or homicide or suicide or automobile crashes?

**Fig. 1:**
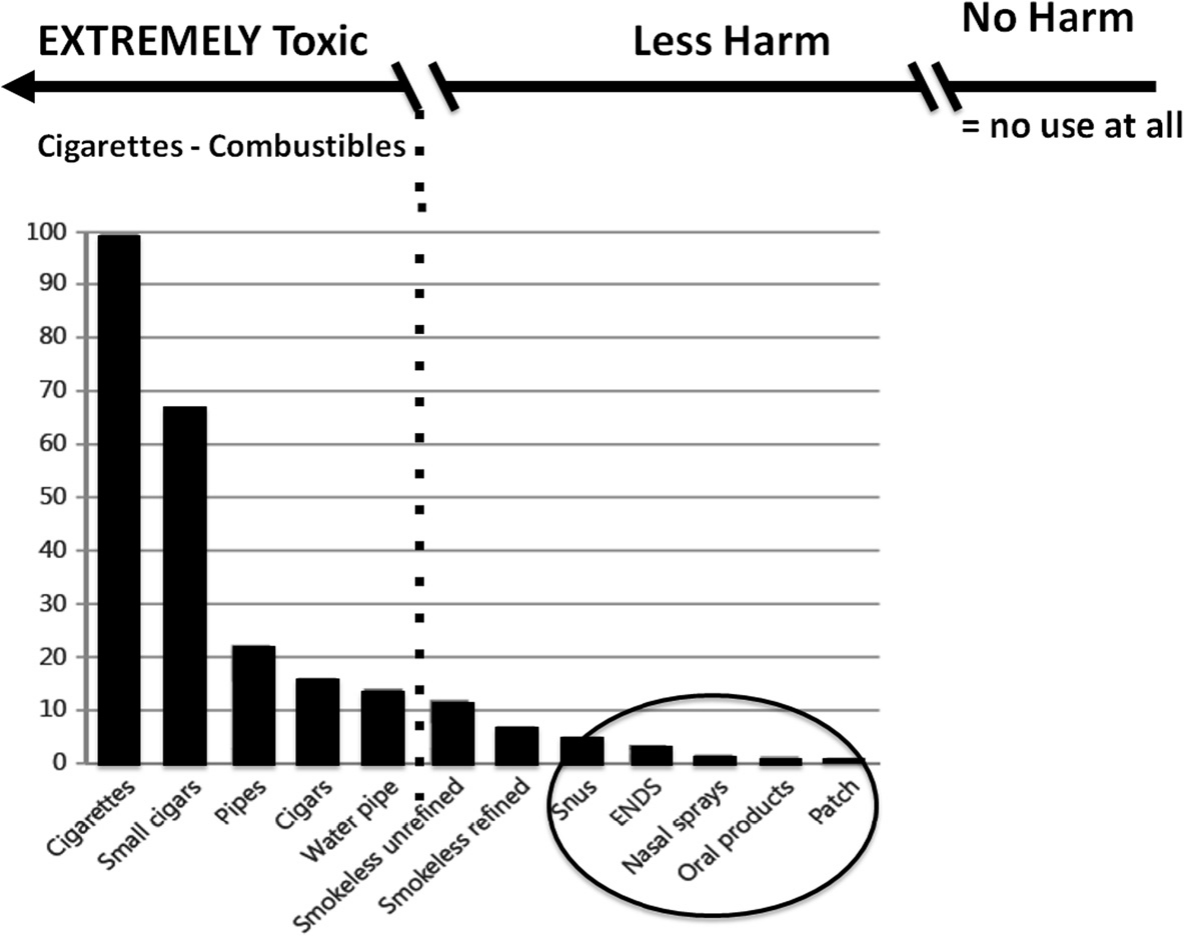
Harm Minimization Continuum (Adapted from Nutt et al 2014 [65])

The credible arguments for the risks of snus or vaping products do not range to the level of highly lethal, but are ranging more at the lower levels of “not safe” (see Fig. 1). The established disease epidemiology for smokeless tobacco products as used in Scandinavia or the U.S. demonstrates that these products are substantially less dangerous than cigarettes [33, 44]. A review of the epidemiological literature on snus concluded, “While smoking substantially increases the risk of cancer and CID [circulatory ischemic disease], any increase from snus use is undemonstrated, and if it exists is probably about 1 % of that from smoking,” [45] and was updated with little change in conclusions [34]. Although ANDS are yet to be regulated to assure consistency and quality control, the more carefully done studies and estimates for the risk from vaping ANDS are also low [35, 46]. While ANDS are not harmless, it seems like hyperbole to argue that ANDS would ever approach the lethality of cigarettes when either is used as intended. Would one argue that there would be more premature deaths from exclusive snus or ANDS use than from alcohol? The CDC estimates the annual deaths from alcohol at about 88,000, [47] compared to all-cause mortality of over 520,000 for cigarettes [37]. Is there an estimate for premature deaths from snus or ANDS use that would come close to being the number of deaths from alcohol? We are unaware of any.

Despite significant epidemiological studies that could provide direct comparative data on all-cause mortality from smokeless tobacco use and cigarette use, [48, 49] it is striking how hard it is to find this direct comparison within the same dataset. In their discussion, the authors of these major reports acknowledge that the risks of smokeless tobacco are “considerably smaller than the risks associated with cigarette smoking,” but express their disagreement that smokeless tobacco be marketed as a less-harmful alternative to smoking; and intentionally they prefer only to compare the risks of smokeless tobacco to the risks of nicotine replacement products [48]. The expressed preference to compare the lower risks among lower risk products is an example of how the prevailing framing ideologies have changed from the days of the 1964 Surgeon General’s Report and *Smoke Signals* [20] when the deadliness of cigarettes was stressed as a matter that set the product apart from all others. This blind spot in the literature suggests that some positions can unintentionally bias thinking in one direction.

### Concerns about gateways, brain damage, and addiction as serious harms?

If the direct chronic disease harm from less-harmful, non-combustible smokeless tobacco/electronic cigarette/ANDS products is substantially less than from cigarettes/combustibles, those interested in *tobacco* control (broadly defined) have moved on to new arguments against tobacco use of any kind. For example, despite the evidence for a common underlying liability model having replaced the unproven gateway theory, possible causal “gateways” have increased in importance despite as yet unproven hypothetical fears that ANDS will lead more youth to become combustible users than otherwise would be the case [50, 51]. The shared liability model indicates that risk taking behaviors are common in adolescents and often travel together so that the first behavior is less important as a gateway but rather is an indicator of shared vulnerability to engage in a variety of risky behaviors regardless of which one came first (for details see [51–54]). Unfortunately, the mere threat of a gateway can create media headlines of earnest concern and regulatory attention although surveillance must monitor the real concern that very high prevalence of experimental use of smokeless or ANDS could possibly result in more uptake and progression to regular smoking than would otherwise have been the case [52]. The importance of any alleged causal gateway effects would, however, depend upon the absolute and relative magnitude of any such effects.

For regulators and for future re-framing purposes, an operational definition of the U.S. Food and Drug Administration (FDA)-mandated public health standard is needed to provide a big picture perspective. For example, a Markov model includes all the trajectories (stocks and flows into and out of specific states) of the different product use patterns by groups within the whole population, both users and non-users [53]. If only 5 % of triers of ANDS or snus were *caused* to become lifetime smokers over and above those who would have become smokers anyway and 10 % of potential and current smokers were displaced from becoming smokers, then the overall net effect on the population is to prevent smoking rather than recruit smoking (i.e., an off-ramp rather than an on-ramp or gateway). But, if the large majority of the entire population of youth became triers (70 or 80 %) of ANDS or snus and were to then go on to regularly smoke cigarettes *because* they had first used these products, then that would indicate a serious concern (but, to date, implausible). No research supports the existence of such an effect [51]. What if 70 % or 80 % of ANDS or snus triers (a) did not move on to cigarettes or (b) would have smoked cigarettes even if they had never tried ANDS or snus? (cf. [54]) That would indicate that the *causal* trajectory issues would be of relatively minor concern under most circumstances. The data on snus is clearest, and in the European review, [33] which seemed motivated to emphasize any evidence for on-ramp effects, concluded, “The Swedish data do not support the hypothesis that smokeless tobacco (i.e., Swedish snus) is a gateway to future smoking.” [33]. In fact over 30 years of experience in Sweden supports that snus has contributed to reductions in mortality from smoking [43].

Note that the gateway hypothesis began with the fear that marijuana would lead to heroin use, and it has not survived as a convincing and current issue and has largely been replaced with a shared vulnerability model [55]. This seems especially clear as marijuana legalization is spreading in the U.S.. The recent trends on use by high school students are, if anything, inconsistent with ANDS looking like a causal gateway to cigarettes [51]. As ANDS trial use (use at least once in the past 30 days) has risen, cigarette use has dropped to historically low levels [56]. Fears of unknown futures, coupled with outmoded 20th century framings need to be rethought, lest they blur the landscape and result in missed opportunities for products which are all legally available to adult consumers and could speed the obsolesce of combusted tobacco use.

Two new arguments have emerged to bolster the older status quo and more extreme (i.e. all or nothing) ideologies of tobacco control. These new arguments depend neither upon the relative harms of different products on mortality nor the concern about a gateway to cigarettes. If not a gateway to cigarettes, some believe that ANDS would be a gateway to severe nicotine addiction, along with concern that nicotine could have substantial irreversible ill-effects on the developing brain or other very severe harms even when decoupled from the deadly smoke of tobacco combustion [57, 58]. Nicotine is not harmless and of course should not be used by pregnant women, just like alcohol, or be used, sold or marketed to minors in any shape or form. But to keep perspective, for anyone already smoking who cannot stop, less harmful delivery modes are considered even NRT use for pregnant women as a last resort. Therefore it seems that nicotine harms should not be exaggerated when legitimate concerns are framed, for example with concerns raised by animal studies but scant human evidence that it causes permanent brain damage when decoupled from all the other toxins in inhaled smoke or that nicotine itself either causes or strongly promotes cancer. There is of course a concern about nicotine from in vitro and animal studies and there is undeniable neuro-adaptation to nicotine as a stimulant, as is the case with any psychoactive chemical [59]. It is too early to assess these arguments and know exactly how they should be integrated into policy at the whole population level. Even if one accepted very strong concerns about nicotine use per se, the much greater health risks from the use of nicotine in deadly cigarette/combustible smoke does still mean that some forms of nicotine use (NRT, snus, ANDS) are much safer than others. The key issue for public health is what amount of unintended consequences can a new tobacco control framework accept if the overall population benefits of less harmful modes of nicotine delivery are largely quite positive?

Nicotine does have also some positive effects on the brain that may explain its attraction and continued use, for example in increasing concentration, enhancing memory and speeding information processing and reducing stress or to alleviate boredom and low energy. Nicotine can for some users be viewed like other similar classes of stimulants used to increase energy and concentration and focus when drowsy, to ameliorate milder forms of ADHD symptoms, or to enhance memory and acute cognitive performance, and thus be quite appealing to those with underlying or predisposing mental health or cognitive vulnerabilities [60, 61]. One could also imagine that adverse drug effects on the developing brain could also be an argument that would be applicable to simple sugars (widely consumed by the very young in cola beverages and chocolate). Significant numbers of youth do engage in marijuana use, alcohol use, are given or take psychotropic prescription medicines for ADHD, anxiety and depression, all of which could be concerning because of ill-effects on the developing brain but where benefits might be judged to outweigh adverse events or side effects under some circumstances. Reviews of the effects on brain maturation include factors like alcohol, nicotine, caffeine, nutrition, gender, stress, and socio-economic status [58, 62]. Vigilant, prudent policies and enforcement of policies is always needed to protect youth from any and all drugs of potential abuse (e.g. opioids and heroin), but frameworks that selectively exaggerate nicotine fears are to be questioned and may do more harm than good in the long run at the whole population level (i.e., for any smokers who may now want to use nicotine in another form of delivery - NRT, smokeless, ANDS) [31, 32, 39, 42, 51, 53, 54].

### A balanced look at absolute and relative harms points to new frameworks for tobacco control

The overview of leading themes here has focused on images and frames that have helped guide tobacco control as it has dealt with recreational tobacco/nicotine products (see Table 1). We have not discussed in any detail the importance of the introduction and promotion of nicotine replacement products in the 1980s and their more recent acceptance for over-the-counter use and long-term use to promote smoking cessation that has no doubt contributed to re-framings related to nicotine [32, 63, 64]. The change that came about when cigarettes were judged to be highly lethal, when used as intended, and more lethal than a sum of other sources of public health harm was a kind of watershed moment in tobacco control. The emergence of reduced harm products like Swedish low nitrosamine snus, NRT, and ANDS raise critical issues regarding the leading themes of the tobacco control field going forward. The “continuum of risk” can be considered an updated framing that has been proposed to help guide tobacco control efforts [65] (see Fig. 1).

Identifying a theme like “the continuum of risk” is not the same as establishing a detailed framework with which to guide tobacco control. The net public health impact of ANDS will be a complex interaction of many factors at multiple levels of influence. Systems thinking and simulation modeling tools will be needed along with more informative data before we will be able to say how best to maximize the benefits of ANDS as a disruptive technology and minimize the hypothetical harms of ANDS to the population as a whole, including users and non-users and especially youth [31, 42, 51, 53]. Regulators and policymakers must keep the big picture in mind when framing key messages to accurately inform consumers.

### “Cigarette control” remains the priority as does the de-normalization of cigarettes/combustibles

The arguments for controlling tobacco/nicotine products should not be uniform across all products, because the risks are not uniform, but dramatically different. If the *Smoke Signals* media handbook [20] were honestly rewritten for snus or ANDS, these products would be seen as among the least risky of popular recreational drug products. When the cigarette control movement learned to oppose the powerful pro-tobacco arguments with evidence-based symbolically-charged responses, it was a large leap forward for cigarette control. When these arguments are misapplied to products that do not approach cigarettes in the damage caused to users and bystanders, it is fallacious, misleading, and compromising to credibility. While it has been feared that ANDS will re-normalize smoking, it could be likelier that the availability of satisfying, much less dangerous cigarette substitutes will act to make it be all the more abnormal for someone to be smoking deadly cigarettes/combustibles.

Those who want to advance tobacco control should appreciate that (a) cigarette/combusted tobacco control remains the highest priority and (b) the arguments against the use of products like vape and snus should not be grounded inappropriately in broad-based all or nothing anti-cigarette arguments. Tobacco control arguments should be proportionate to the absolute and relative harms of each class of products, especially the most deadly combustible products, and be science-based [31, 42]. And, we should work hardest to reduce demand for and the appeal of cigarettes/combustibles [66, 67] which remain highly lethal when used as intended and deadly to more individuals each year than heroin, cocaine, alcohol, AIDS, fires, homicide, suicide and automobile crashes COMBINED. As tobacco control looks to the future, a more complex road map (a framework rather than just an assemblage of themes) is needed to guide arguments, strategies, interventions, and policies to most rapidly eliminate the preventable deaths, inordinate disease burdens, and suffering at the whole population level [53].

It has been said by systems scientists “for every complex problem there is a simple solution … and its wrong.” [68–70]. An integrated and overarching framework is needed within which the complex patterns of poly-tobacco and nicotine use behavior must be viewed [42, 53]. For example, an emerging Markov model framework has been proposed to identify all shifts in the patterns of tobacco use that can alter the ultimate population impact [53]. Given an estimated 1 billion preventable premature deaths worldwide in the 21^st^century, the stakes are enormously high to do more. Sharper, unambiguous themes and messages for different product classes would enhance accurate consumer, policymaker, advocacy and stakeholder knowledge, attitudes, beliefs and actions. Aligned common ground about the relative harms of the different classes of tobacco and nicotine delivery products would more powerfully drive motivated consumer behavior change in the direction of reducing the death and disease burden, overwhelmingly caused by use of lethal combustibles/cigarettes. Leading themes, frames, messages, and slogans all really matter.

## Conclusions

The last 50 years of tobacco control in the U.S. have regularly engaged issues of absolute risk and harm reduction, but have done so in varying ways (see Table 1). The recognition that cigarettes were deadly when used as intended and more lethal than a number of other unsafe products combined was influential and important in the progress of tobacco control. In subsequent years, other forms of tobacco use were treated as similar to cigarettes in issues raised [42]. It is important to make clear distinctions between the classes of tobacco/nicotine products as they differ substantially in risk to the user and to focus tobacco control efforts on reducing the use of cigarettes and other combustible products (see Fig. 1). Complex models [42, 53] should be employed in tobacco control in order to not treat products with large differences in risks as if they are the same [31]. A new reframing of leading themes can align action plans to more powerfully and rapidly achieve population-level benefit and minimize harm. The goal of updating the framing with a new synthesis of management of all forms of nicotine delivery is to eliminate use of the most appealing, addictive and deadly form of tobacco delivery in our lifetime - the smoking of combustible tobacco products - and thus expeditiously prevent the premature deaths of 1 billion people projected to occur worldwide by 2100, if the contentious debate is not resolved.

## Ethics (and consent to participate)

Not applicable.

## Consent to publish

Not applicable.

## Availability of data and materials statement

Not really applicable. Widely available sources were used.

## Abbreviations

ADHD: attention-deficit/hyperactivity disorder
AIDS: acquired immunodeficiency syndrome
ANDS: alternative nicotine delivery system
FDA: United States Food and Drug Administration
FTC: United States Federal Trade Commission
NRT: nicotine replacement therapy
U.S.: United States of America
